# Differentiation of osteosarcoma from osteomyelitis using microarchitectural analysis on panoramic radiographs

**DOI:** 10.1038/s41598-022-16504-9

**Published:** 2022-07-19

**Authors:** Ji-Hun Jung, Kyung-Hoe Huh, Tae-Hoon Yong, Ju-Hee Kang, Jo-Eun Kim, Won-Jin Yi, Min-Suk Heo, Sam-Sun Lee

**Affiliations:** 1 Department of Oral and Maxillofacial Radiology, School of Dentistry and Dental Research Institute, Seoul National University, Daehak-ro 101, Jongro-gu, Seoul, 03080 Korea; 2 Department of Applied Bioengineering, Graduate School of Convergence Science and Technology, Seoul National University, Daehak-ro 101, Jongro-gu, Seoul, 03080 Korea; 3 Department of Oral and Maxillofacial Radiology, Seoul National University Dental Hospital, Daehak-ro 101, Jongro-gu, Seoul, 03080 Korea

**Keywords:** Cancer screening, Oral cancer detection, Cancer imaging, Diseases, Oncology

## Abstract

Diagnosing osteosarcoma (OS) is very challenging and OS is often misdiagnosed as osteomyelitis (OM) due to the nonspecificity of its symptoms upon initial presentation. This study investigated the possibility of detecting OS-induced trabecular bone changes on panoramic radiographs and differentiating OS from OM by analyzing fractal dimensions (FDs) and degrees of anisotropy (DAs). Panoramic radiographs of patients with histopathologically proven OS and OM of the jaw were obtained. A total of 23 patients with OS and 40 patients with OM were enrolled. To investigate whether there was a microarchitectural difference between OS lesions and normal trabecular areas in each patient, two regions of interest (ROIs) were located on the CT images. Three microarchitectural parameters (box-counting FD, fast Fourier transform-based FD, and DA) were calculated. For both OS and OM, significant differences were found for all three microarchitectural parameters. Compared to normal trabecular bone, trabecular bone affected by OS and OM became isotropic and more complex. When comparing OS and OM, a statistically significant difference was found only in DA. Trabecular bones affected by OS became more isotropic than those affected by OM. Microarchitectural analysis, especially DA, could be useful for detecting OS-induced trabecular alterations and differentiating OS from OM.

## Introduction

Osteosarcoma (OS) is the most common primary bone tumor and the third most common malignancy among children and adolescents^1^. OS is defined as a neoplasm that shares the histological finding of osteoid production in association with malignant mesenchymal cells. This tumor is aggressive locally and tends to produce early systemic metastasis^2^.

The current OS treatment is surgical resection with adjuvant chemotherapy. The current five-year survival rate has been reported to be less than 30% in people with metastatic disease, while those who present with nonmetastatic disease experience survival rates exceeding 70%.^3–5^. The detection of OS before metastasis occurs is vital for reducing the extent of surgical resection and improving the survival rate. However, diagnosing bone malignancy is very challenging due to its low incidence and the nonspecificity of its symptoms at presentation^6^. The most common symptoms of OS of the jaw are swelling, pain, tooth mobility, and paresthesia^7,8^. These symptoms could be mistaken for other diseases such as osteomyelitis (OM), which is an infection of the bone. The features of OS and OM are similar on imaging and there are numerous reports of OS being misdiagnosed as OM^9–13^. Panoramic radiographs have frequently been used for imaging of patients in dental clinics. Therefore, panoramic radiographs could be considered as a screening tool for detection of bone malignancies, such as OS. The microarchitecture of the bone, in addition to the bone mass, has been used as a useful predictor of mechanical strength^14–16^. Recently, microarchitectural analysis using panoramic radiographs was used to assess patients with diseases related to bone metabolism, and fractal analysis of the trabecular bone texture was performed in most of these studies^17–21^. The fractal dimension (FD) represents the complexity of the trabecular structure. Measurement of the FD in panoramic radiographs has been applied in various fields, including peri-implant bone assessment, mandibular bone-healing after orthognathic surgery, and trabecular alterations in medication-related osteonecrosis of the jaw^22–25^.

Another important imaging parameter for microarchitectural analysis is anisotropy. An anisotropy evaluation is useful for characterizing the degree of directional organization of the trabecular bone. The degree of anisotropy (DA) represents the degree of trabecular polarization in a particular direction. Several types of bones have been evaluated already for anisotropy, including the hip, calcaneus, vertebrae, and radius^26–28^. Changes in the DA were examined in several studies using the jawbone, and trabecular bone changes in postmenopausal osteoporosis, trabecular bone quality assessment at dental implant sites or as an indicator of implant stability, and condylar internal trabecular bone structure have also been investigted^29–32^.

Despite the recent application of microarchitectural analysis of trabecular bone in the jaw, neither FD nor DA has been used to detect bone malignancies of the jaw. A correct diagnosis of OS is vital and its differention from OM is very challenging based on clinical and imaging features. There has been no study, however, on the microarchitectural analysis of panoramic radiographs for detecting and differentiating OS from OM. We hypothesized that the OS-induced trabecular microarchitecture is different from the normal trabecular architecture and that the OS-induced trabecular microarchitecture is also different from the OM-induced trabecular microarchitecture. The present study aimed to investigate the possibility of detecting OS-induced trabecular bone changes and differentiating them from OM on panoramic radiographs by analyzing FD and DA.

## Results

A total of 23 patients (11 female; 12 male) with OS and 40 patients (21 female; 19 male) with OM were enrolled in the present study. The mean age of the patients with OS was 46.2 years (range 13–83 years) and the mean age of patients with OM was 45.2 years (range 11–68 years). As for the site of occurrence, six patients had OS in the maxilla (26%) and 17 in the mandible (74%), and eight patients had OM (20%) in the maxilla and 32 in the mandible (80%). Comparison of age, sex, and anatomical sites between the OS and OM groups did not show any significant differences (Table 1).

**Table 1:** Summary of the demographic characteristics and sites of occurrence of the 23 subjects with osteosarcoma and 40 subjects with osteomyelitis.

Characteristic	Osteosarcoma (n = 23)	Osteomyelitis (n = 40)	*P-value*
Age			0.82
Mean age	46.2	45.2	
Range	13–83	11–68	
Sex, no. (%)			0.721
Female	11 (48)	21 (52.5)	
Male	12 (52)	19 (47.5)	
Site			0.754
Maxilla	6 (26)	8 (20)	
Mandible	17 (74)	32 (80)	

Age was compared using an independent sample *t*-test and sex and site were compared using a chi-squared test.

The intraclass correlation coefficients indicating interobserver and intraobserver agreement are listed in Table 2. Both intraobserver and interobserver reliabilities for repeated measurements were found to range from good to excellent.

**Table 2:** ICCs of bone microstructural parameters for intraobserver and interobserver agreements.

	Box-counting FD				FFT-based FD				DA			
	OM lesions	OS lesions	Normal areas in OS patients	Normal areas in OM patients	OM lesions	OS lesions	Normal areas in OS patients	Normal areas in OM patients	OM lesions	OS lesions	Normal areas in OS patients	Normal areas in OM patients
Intraobserver ICC	0.864	0.808	0.762	0.780	0.915	0.892	0.758	0.923	0.856	0.801	0.897	0.805
Interobserver ICC	0.835	0.768	0.772	0.753	0.904	0.772	0.816	0.946	0.788	0.840	0.834	0.774

*DA* degree of anisotropy; *FD* fractal dimension; *FFT* fast Fourier transform; *ICC* intraclass correlation coefficient; *OS* osteosarcoma; *OM* osteomyelitis.

Values of box-counting and fast Fourier transform (FFT)-based FDs and DAs satisfied the normal distribution as confirmed through both the Kolmogorov–Smirnov test and the Shapiro–Wilk test. Table 3 shows comparisons between OS and OM groups for box-counting and FFT-based FDs, and DA. There was no statistically significant difference in all the three parameters when comparing the normal areas between OS and OM groups. In each OS and OM group, statistically significant differences between lesions and normal areas were found for all the three microarchitectural parameters (*P* < 0.01). The largest difference between OS lesions and normal areas was shown for DA. Both methods of FD showed higher values in OS lesions than in normal areas (*P* < 0.05). OS lesions demonstrated lower values for DA than those of normal areas (*P* < 0.001). Statistically significant differences between OS and OM lesions were found only for DA. OS lesions had lower values for DA than OM lesions (*P* < 0.001).

**Table 3:** DA and FDs between OS (*n* = 23) and OM (*n* = 40) groups.

	Normal areas in OS patients (mean ± SD)	Normal areas in OM patients (mean ± SD)	*P*-value	OS lesions (mean ± SD)	OM lesions (mean ± SD)	*P*-value
Box-counting FD	1.541 ± 0.085	1.545 ± 0.071	0.808	1.591 ± 0.063	1.611 ± 0.076	0.276
FFT-based FD	2.093 ± 0.140	2.103 ± 0.136	0.778	2.194 ± 0.104	2.179 ± 0.168	0.674
DA	0.822 ± 0.056	0.812 ± 0.042	0.445	0.902 ± 0.042	0.852 ± 0.035	0.001*

*DA* degree of anisotropy; *FD* fractal dimension; *FFT* fast Fourier transform; *OS* osteosarcoma; *OM* osteomyelitis; *SD* standard deviation. Significant differences are indicated by *P < 0.05. Statistical analysis was performed with an independent sample *t*-test. Comparisons between lesions and normal areas in each OS and OM group were performed using a paired *t*-test and showed a significant difference for all the three microarchitectural parameters (*P* < 0.01), not shown in the table.

**Figure 1:**
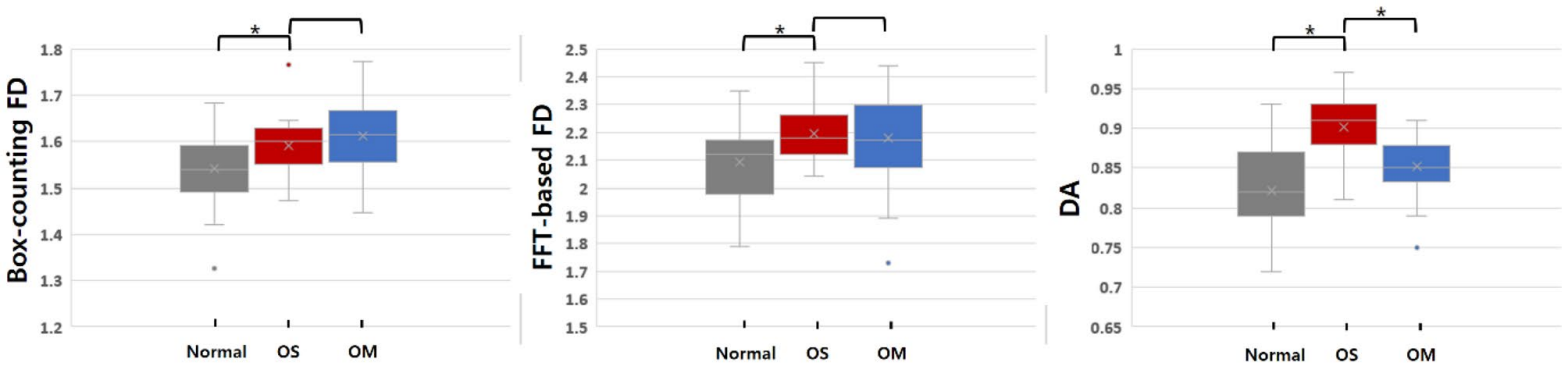
Box plot of the degree of anisotropy (DA), fast Fourier transform (FFT)-based fractal dimension (FD), and box-counting FD. A paired *t*-test was used to compare normal areas and osteosarcoma (OS) lesions. An independent sample *t*-test was performed to compare OS and osteomyelitis (OM). *Significant difference (*P* < 0.05).

Figure 1 shows box plots of the microarchitectural analyses between normal areas, OS, and OM lesions altogether. For OM lesions, as well as for OS lesions, statistically significant differences from normal areas were found for all three microarchitectural parameters. Box-counting and FFT-based FDs did not show a statistically significant difference between OS and OM lesions. However, DA revealed statistically significant differences between OS and OM lesions.

## Discussion

The present study investigated the possibility of detecting OS-induced trabecular bone changes and differentiating them from OM lesions on panoramic radiographs by analyzing the microarchitecture. Significant differences in the trabecular microarchitecture between OS lesions and normal areas were found for the three microarchitectural parameters. Of the three parameters; that is, box-counting FD, FFT-based FD, and DA, the largest difference between OS lesions and normal areas was shown for DA and only DA showed a significant difference between OS and OM lesions.

In the jawbone, the direction of stress applied to the anterior, posterior, and condylar regions are different. Therefore, the trabecular microstructure for each region is different^33,34^. When analyzing the microarchitectural differences between OS lesions and normal areas, the same corresponding anatomical areas of each patient were compared. One region of interest (ROI) was located at the center of OS lesions, and the other ROI was located at the corresponding normal area on the opposite side. Then, the two ROIs were compared. Using this process, we analyzed the differences in the trabecular microstructures between OS lesions and normal areas while controlling for various confounding factors such as age, sex, and anatomical sites. Comparison of age, sex, and anatomical sites between the OS and OM groups revealed no significant differences. In summary, we think that the influence of microarchitectural differences according to age, sex, and anatomical sites could be minimized when comparing OS and OM groups.

The DA is a well-known contributor to bone quality and it is a relatively independent value that has no redundant correlation with other microstructural parameters in the jaw^35^. This is the reason why the DA was considered as a candidate for being used as a microarchitectural discriminator in the present study. Although the DA is usually determined by the ratio between the maximum and minimum radii of the ellipsoid of the mean intercept length^28^, the FFT-based method was used. FD was also calculated using this process in the present study. A strong correlation between these two DA analyses; that is, the mean intercept length method and the FFT-based method, was found in a previous study^33^. A low DA value means that the trabecular bones are structurally isotropic and evenly distributed without an apparent directional inclination. Our results showed that the OS lesions had lower DAs than normal areas. This means that OS lesions had a more isotropic trabecular structure. This can be thought of as the result of haphazard osteolysis and bone formation in OS. Our results also revealed that OS lesions had lower DAs than OM lesions, indicating more isotropic bone structure in OS compared to OM. In a previous study in which DAs were analyzed over time after injecting OS cells into the tibias of mice, OS lesions demonstrated a lower DA value^36^, which is consistent with the results of the present study. In that previous study, micro-CT was used as an imaging modality for DA analysis, and the authors suggested that micro-CT was a complete and fairly accurate method for monitoring OS. However, micro-CT cannot be applied to humans due to radiation exposure. Instead, the possibility of applying DA analysis for the early detection of malignancies using panoramic radiographs, which are widely used in the field of dentistry, is expected to be very high.

It should be noted that in osteoporosis, the trabecular microstructure of the vertebra becomes anisotropic rather than isotropic due to a preferential loss of trabeculae with less mechanical competence^37,38^. Since the increase or decrease in the DA value might vary depending on the kind of diseases and bones being analyzed, further studies on DA analysis using various diseases and bones are needed.

FD is widely used in the microstructural analysis of trabecular bone because FD, in addition to bone mineral density, has been shown to correlate with the biomechanical properties of bone. However, FD might be influenced by many factors, such as noise produced during the imaging process^39,40^ and the method used, so studies analyzing FD should be designed carefully^41^. On the other hand, FDs are known to be insensitive to small variations in x-ray exposure, beam alignment, and the ROI position^42,43^. In the present study, to obtain more reliable results, FD values were calculated using two different methods (box-counting and FFT-based FD). Both methods showed significantly higher values in OS lesions than in normal areas. This means that the complexity of trabecular bone was increased in OS. Other studies using FD analysis revealed that FD was decreased immediately after orthognathic surgery and increased gradually over time^23^ and that FD values of the implant success group were significantly higher than those of the failure group^22^. Like the bone-healing process in orthognathic surgery and implant osseointegration, pathological bone-forming diseases, such as OS and OM, demonstrated an increase in FD values in the present study. However, the present study showed no significant differences in FD values in the comparison between OS and OM. Therefore, it is not be possible to differentiate OS from OM with FD analysis.

With the increasing prevalence of fragility fractures due to osteoporosis, it is vital to diagnose osteoporosis early to prevent this condition. Therefore, many studies have analyzed bone microstructures. On the other hand, studies on the analysis of bone microstructure changes caused by malignancies are rare^44,45^. To our knowledge, the present study is the first to analyze microarchitectural bone changes caused by a malignancy on panoramic radiographs. In a study on OS that occurred in other parts of the body, including the femur, pelvis, and fibula, the rate of misdiagnosis was found to be 23%^46^. In people over the age of 60 years, a misdiagnosis rate of 43% was found due to atypical radiological findings in combination with longer time-lapses from the onset of the first symptoms to the definitive diagnosis^47^. Considering the higher rate of misdiagnosis upon initial presentation^11–13^, we suggest that microarchitectural analysis using panoramic radiographs can help facilitate the early diagnosis of bone-forming sarcomas, such as OS.

The present study has some limitations. First, image noise, which might affect the microarchitectural analysis, is inherent in panoramic radiography. However, in many other studies, microstructural analysis of trabecular bone using panoramic radiographs has been performed successfully and its effectiveness has been proven. We think that image processing with Gaussian blur, erosion, and dilation would be enough to minimize the effects of image noise. Second, OS often occurs as one of three types: osteoblastic, chondroblastic, and fibroblastic. The possibility of different trabecular microstructures according to the OS type was not considered and controlled for in the present study due to the small number of patients with OS. Third, the values of the microarchitectural parameters, such as FD and DA, may vary depending on size of the ROI and where the ROI is placed within a lesion. The effect of ROI size on microarchitectural analysis using the jawbone has yet to be studied.The periphery of OS and OM lesions might show a different microstructure from the center of the lesion. We considered the lesion’s center as the area reflecting the characteristics of the lesion, and ROIs were located at the center of the lesions with reference to the CT image. The reliability of the methods in the present study was also tested. Intraobserver and interobserver agreement was found, which demonstrated good to excellent reproducibility.

The microstructural changes observed in the panoramic radiographs of the jawbone of patients with OS were assessed. The trabecular bone affected by OS became isotropic and more complex compared to normal areas. In addition, the comparison between OS and OM showed more isotropic trabecular changes with OS and similar complexity. Microarchitectural analysis, especially DAs, could be useful in detecting OS-induced trabecular alterations and differentiating OS from OM on panoramic radiographs, which are widely used as an imaging modality in dental clinics.

## Methods

This retrospective study was approved by the Institutional Review Board of the Seoul National University Dental Hospital (IRB066/03-22), and the need for informed consent was waived. All methods were performed in accordance with the relevant guidelines and regulations.

### Patients

Digital panoramic radiographs of patients with histopathologically proven OS of the jaw from January 2000 to December 2020 and OM of the jaw from 2015 to 2020 were obtained from the picture archiving and communicating system at Seoul National University Dental Hospital. Patients who had a history of taking antiresorptive or antiangiogenic agents and who received radiotherapy or chemotherapy before the panoramic radiograph were taken were excluded. Patients with intramedullary OS were included, while those with surface OS were excluded. For comparisons between OS lesions and normal control areas in the same jaw, the patients with OS lesions in the anterior region of the jaw, with no corresponding normal control area on the opposite side, were also excluded. Accordingly, patients with OM lesions in the anterior region of the jaw were excluded from comparisons between OS and OM. General demographic information, including age and sex, was collected from electronic dental records.

### Panoramic radiographs

All of the panoramic radiographs were acquired using the same digital panoramic machine (OP100, Instrumentarium Corp., Tuusula, Finland) at 66–73 kVp, 6.4–12 mA, and with an exposure time of 16.8–17.6 s. Patients were positioned in the panoramic machine according to the manufacturer’s recommendations; the vertical line produced by the machine was aligned with the patient’s sagittal plane, and the Frankfort horizontal plane was parallel to the floor. All images were obtained using PSP image plates (12 × 10 inches) and read by an FCR system (Fuji Computed Radiography 5000R, Fuji Photo Film Co. Ltd., Düsseldorf, Germany). Images were subsequently stored in a BMP format with a matrix of 1976 × 976 pixels, an image file size of 1.83 Mb, and 8-bit gray levels.

### Selection of ROI

**Figure 2:**
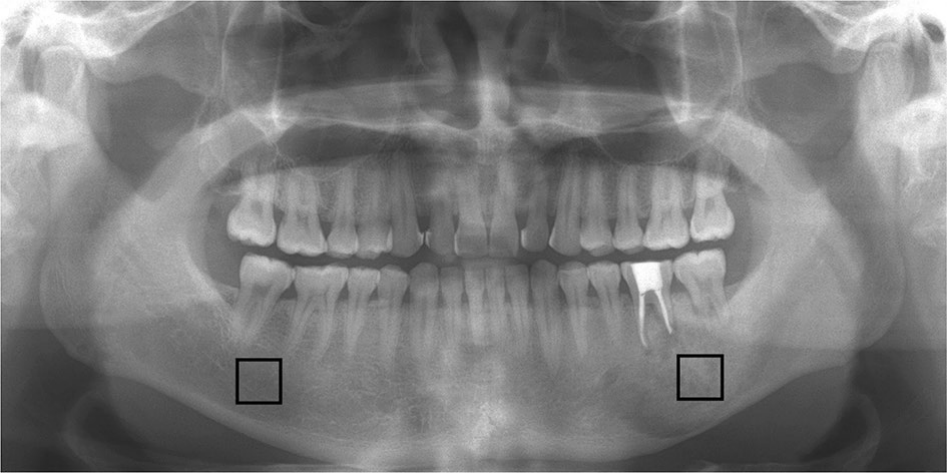
Panoramic radiograph of a 48-year-old man with osteosarcoma (OS) on the left mandible. Note the location of the two square regions of interest (ROIs): one ROI at the center of the OS on the left mandibular body and the other ROI in the corresponding normal trabecular bone on the right mandibular body.

To investigate whether there was a microarchitectural difference between OS lesions and normal trabecular areas in each patient, two ROIs were located on the images: one included the center of the OS lesions and the other was in the corresponding normal trabecular bone on the opposite side (Fig. 2). Both bilateral ROIs were square-shaped and the same size (average size: 96.3 × 96.9 pixels, range of size: 71–127 pixels) in each panoramic image, The ROIs did not include tooth roots. For measurement of FD, a box-counting method and FFT-based method were used. For measurement of DA, directional FD was utilized^33^. Three microarchitectural parameters of box-counting FD, FFT-based FD, and DA in the two bilateral ROIs were analyzed and compared.

**Figure 3:**
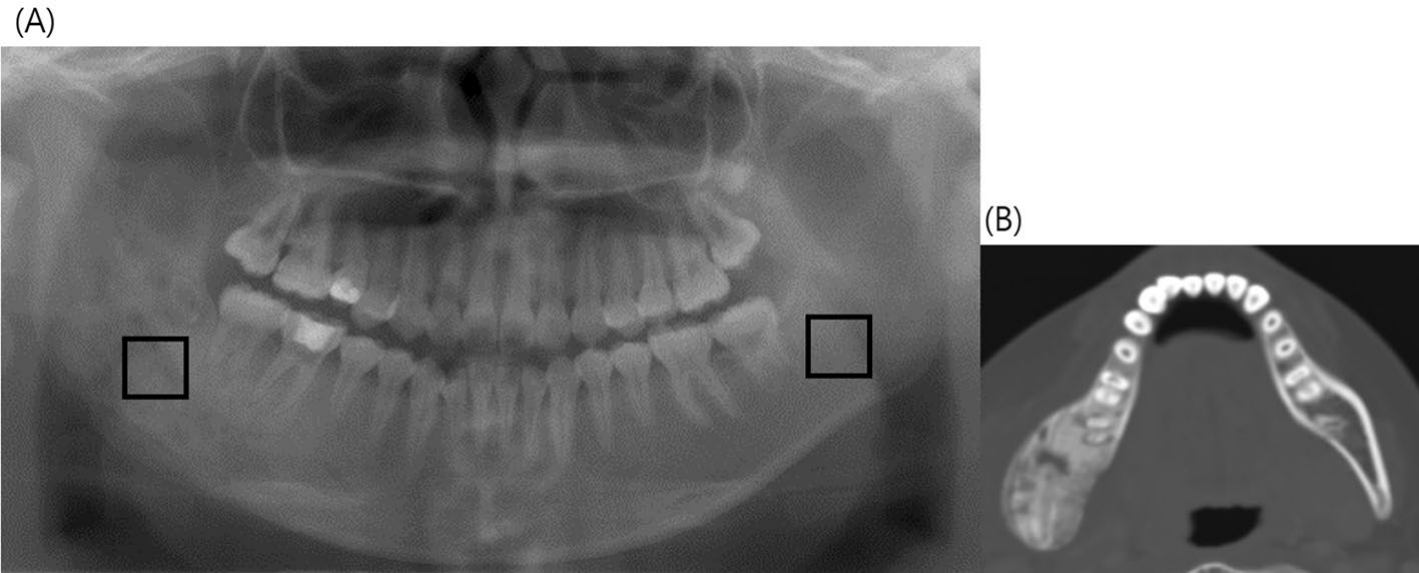
Panoramic radiograph of a 30-year-old woman with osteomyelitis (OM) on the right mandible. (**A**) A ROI was located at the center, or the representative area, of the lesion on a panoramic radiograph with reference to the corresponding CT image. The other ROI was placed in the corresponding normal trabecular bone on the left mandibular body. (**B**) CT image shows the OM lesion of the right posterior mandible more clearly.

For comparison of OS and OM lesions, ROIs were also defined in panoramic images of OM patients. The ROIs of OM lesions were square-shaped (average size: 89.5–91.0) and did not include tooth roots. They were localized in a representative area, mostly at the epicenter of the lesion (Fig. 3). To identify the representive epicenter of a lesion, CT images were used as an aid. To investigate whether there was a microarchitectural difference in normal trabecular areas between OS and OM groups, ROIs were placed bilaterally in panoramic images of OM patients as well. Box-counting FD, FFT-based FD, and DA of the OM lesions were analyzed and compared with those of the OS lesions.

### Calculation of box-counting FD

**Figure 4:**
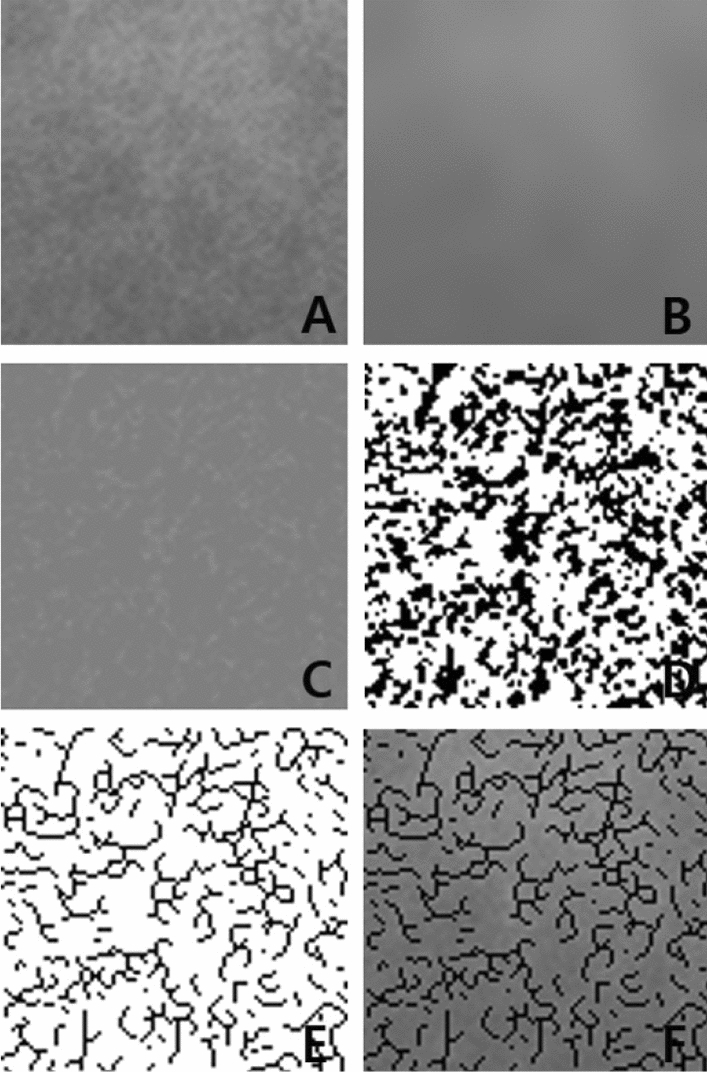
Digital analysis of the trabecular bone morphology. (**A**) A region of interest of the trabecular bone located in the center of the osteosarcoma (OS) area on the left posterior mandible in a patient’s panoramic radiograph in Fig. 1. (**B**) Result after blurring this region. (**C**) Result after subtracting (**B**) from (**A**) and adding 128 (**D**) binary versions of the image (**C**). (**E**) The trabecular pattern is skeletonized. (**F**) Addition of images (**A**) and (**E**) to visually demonstrate that the skeletonized image corresponds to the original trabeculae.

ImageJ (ver.1.52p National Institute of Health, Bethesda, MD, USA) was used to calculate the box-counting FD, which has been a traditional microarchitectural analysis method with two-dimensional (2D) radiographs. Figure 4 shows the procedure for making skeletonized images from a panoramic radiograph. All ROIs were processed using the method reported by White and Rudulph^48^. Briefly, the transferred ROI was filtered using Gaussian blur (sigma 5) to remove the fine and medium scale variations in the image brightness and then saved. The blurred image was then subtracted from the original image, and a gray value of 128 was added. The image was then made binary by threshold at a gray value of 128. The resultant image was eroded and dilated to reduce high-frequency noise. The binary image was outlined and skeletonized. The skeletonized image was used for the calculation of box-counting FD. The widths of the boxes were 2–64 pixels. All digital manipulations and measurements were made within the ROIs.

### Determination of FFT-based FD and DA

The process for calculating the FFT-based FD and DA was carried out in MATLAB R2020a (MathWorks, Natick, Massachusetts, USA)^33,49^. The FFT-based technique was used for calculating the FDs of the 2D radiographs previously^33,39,41,50^. The power spectrum of a local region was converted into the polar coordinate system. The FD was calculated from a curve determined by taking the logarithm of the spectrum versus the logarithm of the frequency. The directional FDs were calculated as a function of orientation based on the Fourier slice theorem (or central slice theorem), which states that the values of a one-dimensional Fourier transform of a parallel projection of an image along a line with the direction were identical to the data along the same line in the 2D Fourier transform of the image^33^.

The polar plot of FDs was constructed by the directional FDs calculated in the directions 0 to 180 degrees. The plot described the moment of inertia of an object as a function of its orientation. To quantify the structural anisotropy of the trabecular bone, the major and minor principal axis directions of inertia were determined by geometrical moments calculated from the polar plot of FDs. The anisotropy was quantified as the ratio of the major and minor axes of the best-fitting ellipse^33^.

**Figure 5:**
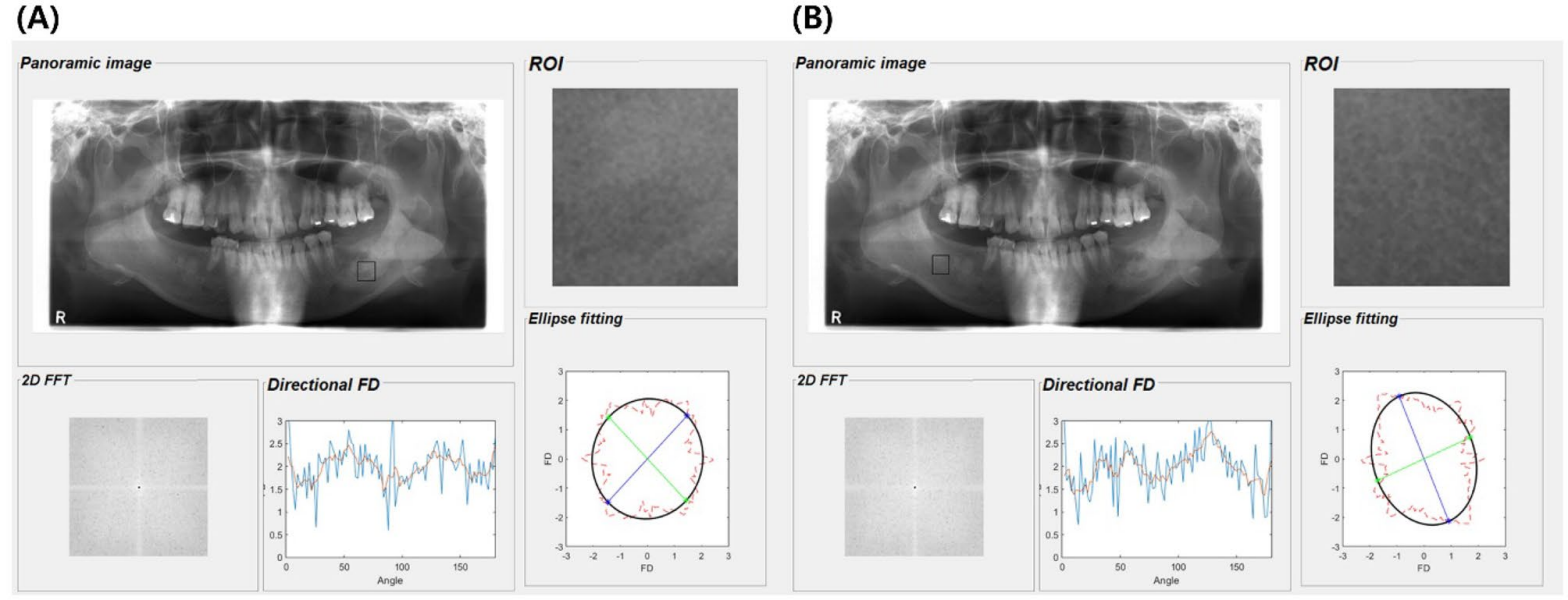
An example of the fast Fourier transform (FFT)-based method of calculating the fractal dimensions (FDs) and degree of anisotropy (DA) of a panoramic radiograph from patients with osteosarcoma (OS). The DA of the region of interest in the OS (**A**) on the left mandible was 0.96 and that of the normal trabecular bone (**B**) on the right mandible was 0.81. OS lesions demonstrated more isotropic trabecular bone structure than the normal area.

In the program, as the measured DA number approached 1, the DA value decreased, and as the measured DA number approached 0, the DA value increased. This meant that the higher the measured DA number, the lower the DA value and the more isotropic the trabecular microarchitecture (Fig. 5).

### Intra- and interobserver reliability

The selection of ROIs and subsequent calculations of FD and DA were performed by two oral and maxillofacial radiologists (KHH, JEK) with over 10 years of experience. Both radiologists had previously been coached with regard to the selection of the ROIs. To quantify intraobserver agreements, the main radiologist (KHH) assessed ROI selections two times with an interval of two weeks between assessments. To test interobserver reliability, another radiologist (JEK) performed the same assessment. The strength of the intraclass correlation coefficient was evaluated as follows: values less than 0.5, between 0.5 and 0.75, between 0.75 and 0.9, and greater than 0.9 are indicative of poor, moderate, good, and excellent reliability, respectively^51^.

### Statistical analysis

To demonstrate the reliability of the measurement process by two radiologists, intraobserver and interobserver reliability of repeated measurements were calculated through intraclass correlation coefficient analysis. The Kolmogorov–Smirnov and Shapiro–Wilk tests were conducted to confirm normal distributions of the data. To compare sex and anatomical sites between OS and OM groups, a chi-squared test was performed at a.05 significance level and age was compared with an independent sample *t*-test at a.05 significance level. Values of box-counting and FFT-based FD and DA between lesions and normal trabecular bone in each OS and OM group were compared using a paired *t*-test at a.05 significance level. Comparisions between OS and OM lesions were performed using an independent sample *t*-test at a.05 significance level. IBM SPSS statistics 23 (SPSS Inc., Chicago, IL, USA) was used for statistical analyses.

### Institutional review board statement

By the Institutional Review Board of Seoul National University Dental Hospital, Dental Life Science Research Institute, ethical review and approval were waived because this study is not a human subject research project specified in the Bioethics and Safety Act (IRB No. ERI21003, Notification of deliberation exemption in February 2021).

### Informed consent statement

By the Institutional Review Board of Seoul National University Dental Hospital, Dental Life Science Research Institute, patient consent was waived because this study is not a human subject research project specified in the Bioethics and Safety Act and that it is practically impossible to obtain the consent of the patient through research, and the risk to them is extremely low as it is a retrospective study that uses existing data.

## Data Availability

The datasets generated during and/or analysed during the current study are available from the corresponding author on reasonable request.
